# Substantial LVSI Is Independently Associated with Para-Aortic Nodal Metastasis in Patients Undergoing Laparoscopic Surgical Staging for Endometrial Cancer

**DOI:** 10.3390/curroncol33070430

**Published:** 2026-07-18

**Authors:** Candost Hanedan, Oğuz Kaan Köksal, Şahin Kaan Baydemir, Neslihan Öztürk, Hande Nur Öncü, Vakkas Korkmaz

**Affiliations:** Department of Gynecologic Oncology, Ankara Etlik City Hospital, Ankara 06170, Turkey; oguzkaan134@gmail.com (O.K.K.); drkaanbydmr@gmail.com (Ş.K.B.); neslihan_oztrk@hotmail.com (N.Ö.); handekayahan@gmail.com (H.N.Ö.); korkmazvakkas@gmail.com (V.K.)

**Keywords:** endometrial cancer, substantial LVSI, para-aortic lymph node metastasis, laparoscopic lymphadenectomy, nodal dissemination

## Abstract

Lymphovascular space invasion (LVSI) is a well-established prognostic factor in endometrial cancer; however, its independent contribution to para-aortic nodal metastasis remains incompletely defined. Identifying factors associated with nodal dissemination is important for surgical staging and postoperative management. This retrospective, single-center cohort study included 121 patients with endometrial cancer who underwent laparoscopic pelvic and para-aortic lymphadenectomy. The cohort was predominantly obese (median BMI: 32 kg/m^2^). Clinicopathological variables were analyzed using univariable and multivariable logistic regression models to identify associations with lymph node metastasis and para-aortic lymph node metastasis. Lymph node metastasis was observed in 16.5% of patients, including para-aortic involvement in 9.9%. In univariable analysis, the LVSI category was significantly associated with lymph node metastasis (*p* = 0.002), with substantial LVSI being present in 50.0% of patients with nodal metastasis compared with 14.9% of those without. LVSI was categorized as negative, focal, or substantial. In multivariable logistic regression analyses using negative LVSI as the reference category, substantial LVSI remained independently associated with both overall nodal metastasis (OR 5.44, 95% CI 1.67–17.65, *p* = 0.005) and para-aortic nodal metastasis (OR 6.98, 95% CI 1.81–26.78, *p* = 0.005), whereas focal LVSI was not significantly associated with either outcome. These findings suggest that the extent of LVSI may be relevant to nodal metastasis, with substantial LVSI showing a stronger association than focal LVSI.

## Simple Summary

Endometrial cancer is one of the most common gynecologic cancers, and the spread of cancer cells to lymph nodes is associated with worse outcomes. Identifying patients at risk for lymph node involvement, especially in the para-aortic region, remains challenging because current imaging methods and intraoperative assessments are limited. Lymphovascular space invasion (LVSI), a pathological finding showing cancer cells within blood or lymphatic vessels, is known to be associated with more aggressive disease. However, the association between the extent of LVSI and para-aortic lymph node metastasis has received less attention. In this study, we analyzed 121 patients with endometrial cancer who underwent laparoscopic surgical staging. The cohort was predominantly obese, with a median BMI of 32 kg/m^2^. We found that substantial LVSI was independently associated with both pelvic and para-aortic lymph node metastasis. These results suggest that evaluating the extent of LVSI in routine pathology reports may help identify patients at higher risk and support more personalized postoperative treatment decisions.

## 1. Introduction

Endometrial cancer is the most common gynecologic malignancy in developed countries, with an increasing incidence largely attributed to rising obesity rates [1]. Although most patients present with early-stage disease confined to the uterus, lymph node metastasis remains a key determinant of prognosis and is associated with reduced survival, directly influencing adjuvant treatment decisions [2,3].

While the therapeutic value of lymphadenectomy has been debated [4,5,6], the SEPAL study demonstrated a survival benefit of combined pelvic and para-aortic lymph node dissection in intermediate and high-risk patients, suggesting a role beyond surgical staging [7]. Sentinel lymph node mapping has emerged as a less invasive alternative endorsed by current NCCN guidelines; however, systematic lymphadenectomy remains relevant when high-risk features are present or sentinel mapping is not feasible [8].

Lymphovascular space invasion (LVSI) is one of the factors strongly associated with nodal dissemination in endometrial cancer and has been consistently associated with lymph node metastasis, recurrence, and reduced survival [9,10,11]. Beyond its presence, increasing evidence suggests that the extent of LVSI is a critical determinant of its clinical impact, with substantial LVSI conferring significantly worse oncologic outcomes compared with focal or absent LVSI [3,10]. This distinction has been incorporated into contemporary risk stratification systems and clinical guidelines, including the ESGO/ESTRO/ESP recommendations, as well as the 2023 FIGO staging classification [12,13].

Despite its prognostic importance, the clinical utility of LVSI in surgical decision-making remains limited, as it cannot be reliably assessed intraoperatively. Frozen section analysis lacks sufficient accuracy, and the microcystic, elongated, and fragmented (MELF) invasion pattern may mimic true lymphovascular involvement, leading to potential misclassification. In addition, interobserver variability in LVSI assessment has been reported even among experienced pathologists [14,15,16]. Consequently, intraoperative decision-making is primarily guided by clinicopathological criteria rather than LVSI status.

The Mayo Clinic algorithm, which recommends lymphadenectomy in patients with tumor size ≥2 cm, grade 3 histology, or myometrial invasion ≥50%, remains one of the most widely used approaches for surgical staging based on frozen section evaluation [17]. However, this strategy has inherent limitations, particularly in identifying patients with occult nodal disease who may not meet these criteria but still harbor lymphatic spread. Preoperative imaging modalities also have important limitations: magnetic resonance imaging (MRI) demonstrates a sensitivity of approximately 44% for detecting lymph node metastasis, while positron emission tomography-computed tomography (PET-CT) carries a false-negative rate of up to 54%, rendering both insufficient to reliably exclude nodal involvement, especially micrometastatic disease [18,19,20].

Although the association between LVSI and nodal metastasis in endometrial cancer is well recognized, the clinical importance of distinguishing substantial from focal LVSI in relation to para-aortic nodal metastasis has received relatively little attention. Considering the extent of LVSI may improve postoperative risk stratification and help identify patients at increased risk of para-aortic nodal involvement.

Therefore, the present study aimed to determine whether substantial LVSI is independently associated with para-aortic lymph node metastasis in patients with endometrial cancer undergoing laparoscopic systematic lymphadenectomy and to evaluate its role relative to established clinicopathological risk factors in a real-world surgical cohort.

## 2. Patients and Methods

### 2.1. Study Design

This retrospective, single-center cohort study was conducted at Ankara Etlik City Hospital and approved by the institutional review board (BADEK, Approval No: 20224-0672, Date: 25 September 2024). The requirement for informed consent was waived due to the retrospective design.

Between April 2023 and October 2025, a total of 513 patients who underwent surgical staging for gynecologic malignancies at our institution were screened. Among them, 121 patients with histologically confirmed endometrial cancer who underwent standardized laparoscopic pelvic and para-aortic lymphadenectomy met the inclusion criteria and were included in the final analysis.

Patients were excluded based on the following predefined criteria: non-endometrial malignancies, laparotomic surgical approach, or incomplete pathological data, defined as missing LVSI status or nodal count. Molecular subtype was assessed where tissue was sufficient; two patients in whom immunohistochemical surrogates could not be evaluated due to insufficient tissue were retained and categorized as ‘not assessed’.

As a tertiary referral center, patients undergoing systematic lymphadenectomy are typically selected based on preoperative and intraoperative risk stratification, which may result in enrichment of intermediate- and high-risk cases. This should be considered when interpreting the generalizability of the findings.

### 2.2. Surgical Technique

All procedures were performed laparoscopically by experienced gynecologic oncology surgeons in accordance with a standardized institutional lymphadenectomy protocol. Patients were positioned in the lithotomy position with a slight Trendelenburg tilt.

Uterine manipulator use was at the discretion of the operating surgeon, and both manipulator and non-manipulator approaches were included, reflecting real-world surgical practice.

A total of six trocars were used: one 10 mm umbilical or supraumbilical port, two right and two left 5 mm pararectal ports, and one 10 mm suprapubic port.

Pelvic lymphadenectomy included systematic bilateral removal of external iliac, internal iliac, obturator, and common iliac lymph nodes. Para-aortic lymphadenectomy was performed up to the level of the renal vessels and included the right paracaval, interaortocaval, and left para-aortic compartments.

All lymph node specimens were retrieved in an endobag and extracted transvaginally following hysterectomy.

### 2.3. Pathological Assessment

All specimens were evaluated by dedicated gynecologic pathologists. Histological subtype was classified according to the World Health Organization (WHO) classification. Lymphovascular space invasion (LVSI) was categorized as negative, focal (1–4 involved vessels), or substantial (≥5 involved vessels), based on semiquantitative criteria widely used in contemporary literature and incorporated into current clinical guidelines [12,13]. LVSI assessment was primarily performed on routinely processed hematoxylin and eosin (H&E)-stained sections. When the diagnosis was considered equivocal, additional endothelial immunohistochemical staining (e.g., D2-40 or CD31) was performed at the discretion of the pathologist to confirm true lymphovascular invasion. For statistical analyses, LVSI was treated as a three-level categorical variable (negative, focal, and substantial), with negative LVSI serving as the reference category. Myometrial invasion was classified as <50% or ≥50%, and tumor grade was assessed for endometrioid carcinomas.

Molecular classification was approximated using immunohistochemical surrogate markers and categorized as mismatch repair-deficient (MMRd), p53 abnormal (p53abn), and no specific molecular profile (NSMP). POLE mutation analysis was not routinely performed due to cost and technical limitations and was therefore not available for all patients. Consequently, molecular classification in this study does not represent a complete TCGA-based classification, and tumors categorized as NSMP may include a small proportion of POLE-mutated cases, potentially leading to misclassification. These findings should therefore be interpreted with this limitation in mind.

### 2.4. Statistical Analysis

Continuous variables are presented as median values with corresponding ranges. Differences between groups were assessed using the Mann–Whitney U test. Categorical variables are summarized as frequencies and percentages and were compared using Pearson’s chi-square test or Fisher’s exact test, as appropriate. To identify factors associated with lymph node metastasis and para-aortic nodal metastasis, all clinicopathological variables were initially evaluated using univariable logistic regression analysis. Variables with a *p* value < 0.10 in the univariable analysis, or those considered clinically relevant based on prior literature, were subsequently entered into a multivariable logistic regression model. Model fit was assessed using Akaike’s information criterion (AIC) and pseudo-R^2^ measures. Because of the limited number of outcome events, the number of variables entered into the multivariable models was restricted, and results were interpreted cautiously in light of the low events-per-variable ratio (EPV approximately 5–6). A *p* value of <0.05 was considered statistically significant. All analyses were performed using IBM SPSS Statistics, Version 31.0.2.0 (IBM Corp., Armonk, NY, USA).

## 3. Results

A total of 121 patients with endometrial cancer who underwent laparoscopic pelvic and para-aortic lymphadenectomy were included in the study. The median age of the cohort was 63 years (range 34–80), and the median BMI was 32 kg/m^2^ (range 19–47). The median CA-125 level was 14 U/mL (range 3–329), and the median tumor size was 50 mm (range 5–170). Endometrioid histology was the most common subtype, accounting for 74.4% of cases. Regarding molecular classification, the most frequent subtype was NSMP (57.0%), followed by MMRd (21.5%) and p53-abnormal tumors (19.8%). Deep myometrial invasion (≥1/2) was observed in 59.5% of patients, and substantial LVSI was present in 20.7% of cases (Table 1).

The mean total number of retrieved lymph nodes was 29.3 ± 11.7, including a mean of 17.6 ± 7.9 pelvic lymph nodes and 11.8 ± 6.8 para-aortic lymph nodes. Lymph node metastasis was detected in 20 patients (16.5%). Pelvic lymph node metastasis was present in 14 patients (11.6%), and para-aortic lymph node metastasis was detected in 12 patients (9.9%) (Table 1).

In the univariate analysis evaluating factors associated with lymph node metastasis, substantial LVSI was significantly more frequent in patients with nodal metastasis compared with those without metastasis (50.0% vs. 14.9%, *p* = 0.002). Other variables, including age, BMI, CA-125 level, tumor size, histological subtype, molecular subtype, tumor grade, myometrial invasion, serosal involvement, cervical invasion, and ovarian involvement, were not significantly associated with lymph node metastasis (Table 2).

Multivariable logistic regression analysis identified substantial LVSI as independently associated with lymph node metastasis (OR 5.44, 95% CI 1.676–17.656, *p* = 0.005). Focal LVSI, deep myometrial invasion, BMI, and age were not significantly associated with lymph node metastasis in the multivariable model (Table 3).

When factors associated with para-aortic lymph node metastasis were evaluated, univariate analysis demonstrated that substantial LVSI was significantly more common in patients with para-aortic metastasis than in those without (58.3% vs. 16.5%, *p* = 0.003). Other variables, including age, BMI, CA-125 level, tumor size, histological subtype, molecular subtype, tumor grade, myometrial invasion, serosal involvement, cervical invasion, and ovarian involvement, were not significantly associated with para-aortic lymph node metastasis (Table 4).

In the multivariable logistic regression analysis, substantial LVSI remained independently associated with para-aortic lymph node metastasis (OR 6.98, 95% CI 1.818–26.786, *p* = 0.005). Focal LVSI and CA-125 levels were not significantly associated with para-aortic lymph node metastasis in the adjusted model (Table 5).

In supplementary analyses using two-category LVSI classifications, substantial LVSI remained independently associated with both overall nodal metastasis and para-aortic nodal metastasis when compared with negative/focal LVSI. Consistent associations were also observed when any LVSI was compared with negative LVSI (Supplementary Table S1).

The distribution of pelvic and para-aortic nodal involvement according to LVSI category is presented in Table 6. Among the six patients with isolated para-aortic nodal metastasis (PLN−/PAN+), three (50.0%) had substantial LVSI, whereas none had focal LVSI. Given the limited number of isolated para-aortic events, these findings should be interpreted descriptively.

## 4. Discussion

The present study demonstrates that substantial LVSI is independently associated with both pelvic and para-aortic lymph node metastasis and is independently associated with nodal dissemination in multivariable analyses, with odds ratios of 5.44 and 6.98, respectively. Taken together, these findings suggest that the extent of LVSI, rather than its mere presence, may be an important factor in identifying patients at risk for nodal dissemination, including para-aortic involvement [3,9,10,21].

LVSI represents an early step in the metastatic cascade, reflecting tumor cell dissemination through lymphatic and vascular channels [9,10]. Its presence has been consistently associated with lymph node metastasis, recurrence, and reduced survival, as well as with other high-risk clinicopathological features [3,9,22,23]. Importantly, the extent of LVSI appears to determine its clinical impact. Substantial LVSI has been linked to significantly worse oncologic outcomes, whereas focal LVSI often behaves similarly to LVSI-negative disease [3,10,24,25]. This distinction has led to the incorporation of LVSI extent into contemporary risk stratification systems and the 2023 FIGO staging classification [12,13].

Our findings are consistent with prior studies identifying LVSI as independently associated with nodal metastasis [11,26]. Tatar et al. reported hazard ratios of 10.3 and 18.8 for pelvic and para-aortic nodal involvement, respectively, in a single-center cohort, values directionally comparable to the odds ratios observed in the present study [26]. Similarly, Li et al. demonstrated that substantial LVSI was associated with worse progression-free survival, while focal LVSI showed outcomes comparable to LVSI-negative disease [10]. Furthermore, large multicenter and molecularly stratified analyses have confirmed that LVSI retains its prognostic impact across different molecular subgroups [9,27].

However, most previous studies have primarily focused on pelvic nodal disease, and the independent contribution of substantial LVSI to para-aortic metastasis has remained insufficiently defined [21,28]. In this context, our findings extend the existing evidence by demonstrating that substantial LVSI is independently associated with para-aortic nodal metastasis, thereby highlighting its relevance beyond pelvic disease.

Our cohort was predominantly obese (median BMI: 32 kg/m^2^), and obesity is associated with increased morbidity following lymphadenectomy [29]. Although LVSI cannot reliably guide intraoperative decision-making, it may provide useful information for postoperative risk assessment and help identify patients at increased risk for occult para-aortic disease who may benefit from tailored adjuvant treatment strategies.

Preoperative imaging and intraoperative assessment remain limited, as MRI and PET-CT have suboptimal sensitivity for nodal disease, and LVSI cannot be reliably assessed on frozen section [14,18,19,20]. Consequently, substantial LVSI may provide useful information for postoperative risk assessment and individualized management. Among the six patients with isolated para-aortic nodal metastasis and no pelvic nodal involvement, three had substantial LVSI. Although the small number of cases precludes meaningful statistical analysis, this observation raises the possibility that the extent of LVSI may be associated with para-aortic dissemination even in the absence of pelvic nodal metastasis. This finding should be explored further in larger prospective studies.

This study has several limitations. Its retrospective, single-center design may limit generalizability and introduce selection bias. The relatively small sample size and limited number of nodal metastasis events may have reduced statistical power. In addition, the low events-per-variable ratio should be considered when interpreting the multivariable findings. Given the limited number of para-aortic nodal events (n = 12), pelvic nodal status was not included as an additional covariate in the multivariable model for para-aortic metastasis to minimize the risk of model overfitting and unstable effect estimates. Table 6 presents the distribution of pelvic and para-aortic nodal status according to the LVSI category.

Molecular classification was based on immunohistochemical surrogates without POLE mutational analysis, and survival outcomes could not be robustly assessed due to short follow-up. Furthermore, patients underwent surgery both with and without a uterine manipulator. Although the potential impact of uterine manipulator use on LVSI assessment remains controversial, our retrospective study was neither designed nor sufficiently powered to compare LVSI rates according to manipulator use. Therefore, uterine manipulator use should be considered a potential source of bias and warrants further evaluation in larger prospective studies.

## 5. Conclusions

In conclusion, substantial lymphovascular space invasion was independently associated with para-aortic nodal metastasis in patients with endometrial cancer undergoing laparoscopic systematic lymphadenectomy. These findings suggest that the extent of LVSI may be relevant to its association with nodal metastasis, as focal LVSI was not significantly associated with either outcome. This distinction is particularly relevant in obese patients, where minimizing unnecessary surgical morbidity while ensuring adequate oncologic staging remains a critical clinical challenge. Substantial LVSI may serve as a clinically meaningful postoperative marker to identify patients at increased risk for occult para-aortic disease and inform individualized adjuvant treatment strategies. Further prospective studies with larger cohorts are warranted to validate these findings.

## Figures and Tables

**Table 1:** Baseline clinicopathological and surgical characteristics of the study cohort (N = 121).

Baseline Characteristics	Total (N = 121)
**Age, years, median (range)**	63 [34–80]
**BMI, kg/m^2^, median (range)**	32 [19–47]
**CA-125, U/mL, median (range)**	14 [3–329]
**Tumor size, mm, median (range)**	50 [5–170]
**Histological subtype, n (%)**	
Endometrioid	90 (74.4%)
Serous	16 (13.2%)
Mixed	6 (5.0%)
Clear cell	3 (2.5%)
Carcinosarcoma	3 (2.5%)
Others †	3 (2.5%)
**Molecular subtype (IHC), n (%)**	
NSMP	69 (57.0%)
MMRd	26 (21.5%)
p53abn	24 (19.8%)
Not assessed ‡	2 (1.7%)
Tumor grade, n (%) *	
Grade 1	54 (60.0%)
Grade 2	26 (28.9%)
Grade 3	10 (11.1%)
**Myometrial invasion, n (%)**	
No invasion	6 (5.0%)
<1/2	43 (35.5%)
≥1/2	72 (59.5%)
**LVSI, n (%)**	
Negative	82 (67.8%)
Focal	14 (11.6%)
Positive (substantial)	25 (20.7%)
**Serosal involvement, n (%)**	
Negative	118 (97.5%)
Positive	3 (2.5%)
**FIGO stage [2023]**, **n (%)**	
Stage I	48 (39.7%)
Stage II	46 (38.0%)
Stage III	24 (19.8%)
Stage IV	3 (2.5%)
**Lymph node dissection**	
Total LN retrieved, mean ± SD	29.3 ± 11.7
Pelvic LN retrieved, mean ± SD	17.6 ± 7.9
Para-aortic LN retrieved, mean ± SD	11.8 ± 6.8
**Lymph node metastasis, n (%)**	
Any LN metastasis	20 (16.5%)
Pelvic LN metastasis	14 (11.6%)
Para-aortic LN metastasis	12 (9.9%)
**Additional pathological findings, n (%)**	
Omentum involvement	3/102 (2.9%)
Positive peritoneal cytology	0/121 (0.0%)

* Grade reported for endometrioid histology only (n = 90). † Others include mucinous (n = 1), dedifferentiated (n = 1), and mesonephric-like (n = 1) carcinomas. ‡ Molecular subtype could not be assessed in 2 patients. IHC: immunohistochemistry; NSMP: no specific molecular profile; MMRd: mismatch repair-deficient; p53abn: p53 abnormal; LN: lymph node; LVSI: lymphovascular space invasion; SD: standard deviation; FIGO: International Federation of Gynecology and Obstetrics.

**Table 2:** Univariate analysis of factors associated with lymph node metastasis.

Variable	LN Negative (n = 101)	LN Positive (n = 20)	*p* Value
**Continuous variables**			
Age, years, median (range)	63 [34–79]	61 [40–80]	0.099
BMI, kg/m^2^, median (range)	32 [19–47]	30 [22–42]	0.113
CA-125, U/mL, median (range)	13 [3–329]	20 [3–203]	0.207
Tumor size, mm, median (range)	50 [5–170]	53 [10–140]	0.810
**Categorical variables**			
**Histological type**			0.856
Endometrioid	74 (73.3%)	16 (80.0%)	
Non-endometrioid	27 (26.7%)	4 (20.0%)	
**Molecular subtype (IHC)**			0.306
NSMP	58 (57.4%)	11 (55.0%)	
MMRd	19 (18.8%)	7 (35.0%)	
p53abn	22 (21.8%)	2 (10.0%)	
Not assessed	2 (2.0%)	0 (0.0%)	
Tumor grade *			0.329
Grade 1	46 (62.2%)	8 (50.0%)	
Grade 2	19 (25.7%)	7 (43.8%)	
Grade 3	9 (12.2%)	1 (6.3%)	
**Myometrial invasion**			0.105
No invasion/<1/2	45 (44.6%)	4 (20.0%)	
≥1/2	56 (55.5%)	16 (80.0%)	
**LVSI**			0.002 *
Negative	74 (73.3%)	8 (40.0%)	
Focal	12 (11.9%)	2 (10.0%)	
Positive (substantial)	15 (14.9%)	10 (50.0%)	
**Serosal involvement**			0.435
Negative	98 (97.0%)	20 (100.0%)	
Positive	3 (3.0%)	0 (0.0%)	
**Cervical invasion**			0.323
Negative	85 (84.2%)	15 (75.0%)	
Positive	16 (15.8%)	5 (25.0%)	
**Ovarian involvement**			0.427
Negative	99 (98.0%)	19 (95.0%)	
Positive	2 (2.0%)	1 (5.0%)	

* *p* < 0.05. Mann–Whitney U test was used for continuous variables. Chi-square or Fisher’s exact test was used for categorical variables. Grade reported for endometrioid histology only (n = 90). LN: lymph node; LVSI: lymphovascular space invasion; IHC: immunohistochemistry; NSMP: no specific molecular profile; MMRd: mismatch repair-deficient; p53abn: p53 abnormal.

**Table 3:** Multivariable logistic regression analysis for factors associated with lymph node metastasis.

Variable	OR	95% CI	*p* Value
**LVSI**			
Positive (substantial) vs. Negative	5.44	1.676–17.656	0.005 *
Focal vs. Negative	1.69	0.303–9.430	0.549
Myometrial invasion (≥1/2 vs. <1/2)	2.56	0.760–8.635	0.129
BMI (per 1 kg/m^2^ increase)	1.08	0.970–1.209	0.158
Age (per 1 year increase)	1.01	0.947–1.082	0.717

* *p* < 0.05. OR: odds ratio; CI: confidence interval; LVSI: lymphovascular space invasion. Model fit: McFadden R^2^ = 0.152, AIC = 104 (N = 121). Due to limited events per variable (EPV = 5.0), results should be interpreted with caution.

**Table 4:** Univariate analysis of factors associated with para-aortic lymph node metastasis.

Variable	PA LN Negative (n = 109)	PA LN Positive (n = 12)	*p* Value
**Continuous variables**			
Age, years, median (range)	63 [34–80]	59 [40–74]	0.200
BMI, kg/m^2^, median (range)	32 [19–47]	31 [22–40]	0.876
CA-125, U/mL, median (range)	13 [3–329]	28 [3–203]	0.092
Tumor size, mm, median (range)	50 [5–170]	58 [10–140]	0.476
**Categorical variables**			
**Histological type**			0.670
Endometrioid	81 (74.3%)	9 (75.0%)	
Non-endometrioid	28 (25.7%)	3 (25.0%)	
**Molecular subtype (IHC)**			0.576
NSMP	62 (56.9%)	7 (58.3%)	
MMRd	22 (20.2%)	4 (33.3%)	
p53abn	23 (21.1%)	1 (8.3%)	
Not assessed	2 (1.8%)	0 (0.0%)	
Tumor grade *			0.161
Grade 1	51 (63.0%)	3 (33.3%)	
Grade 2	21 (25.9%)	5 (55.6%)	
Grade 3	9 (11.1%)	1 (11.1%)	
**Myometrial invasion**			0.446
No invasion/<1/2	46 (42.2%)	3 (25.0%)	
≥1/2	63 (57.8%)	9 (75.0%)	
**LVSI**			0.003 *
Negative	78 (71.6%)	4 (33.3%)	
Focal	13 (11.9%)	1 (8.3%)	
Positive (substantial)	18 (16.5%)	7 (58.3%)	
**Cervical invasion**			0.124
Negative	92 (84.4%)	8 (66.7%)	
Positive	17 (15.6%)	4 (33.3%)	
**Ovarian involvement**			0.169
Negative	107 (98.2%)	11 (91.7%)	
Positive	2 (1.8%)	1 (8.3%)	

* *p* < 0.05. Mann–Whitney U test was used for continuous variables. Chi-square or Fisher’s exact test was used for categorical variables. Grade reported for endometrioid histology only (n = 90). PA LN: para-aortic lymph node; LVSI: lymphovascular space invasion; IHC: immunohistochemistry; NSMP: no specific molecular profile; MMRd: mismatch repair-deficient; p53abn: p53 abnormal.

**Table 5:** Multivariable logistic regression analysis for factors associated with para-aortic lymph node metastasis.

Variable	OR	95% CI	*p* Value
**LVSI**			
Positive (substantial) vs. Negative	6.98	1.818–26.786	0.005 *
Focal vs. Negative	0.88	0.064–12.044	0.922
CA-125 (per 1 U/mL increase)	1.008	0.997–1.020	0.154

* *p* < 0.05. OR: odds ratio; CI: confidence interval; LVSI: lymphovascular space invasion; CA-125: cancer antigen 125. Model fit: McFadden R^2^ = 0.144, Nagelkerke R^2^ = 0.187, AIC = 74.8 (N = 121). Due to limited events per variable (EPV = 6.0), results should be interpreted with caution.

**Table 6:** Distribution of pelvic and para-aortic lymph node status and LVSI category within each subgroup.

Nodal Status Group	N (%)	LVSI Negative	LVSI Focal	LVSI Substantial
PLN−/PAN−	101 (83.5%)	74 (73.3%)	12 (11.9%)	15 (14.9%)
PLN+/PAN−	8 (6.6%)	4 (50.0%)	1 (12.5%)	3 (37.5%)
PLN−/PAN+	6 (5.0%)	3 (50.0%)	0 (0.0%)	3 (50.0%)
PLN+/PAN+	6 (5.0%)	1 (16.7%)	1 (16.7%)	4 (66.7%)
Total	121 (100%)	82 (67.8%)	14 (11.6%)	25 (20.7%)

PLN: pelvic lymph node. PAN: para-aortic lymph node. LVSI: lymphovascular space invasion. Percentages in the N (%) column and Total row represent the proportion of the total cohort (N = 121). Percentages in the LVSI Negative/Focal/Substantial columns for the four subgroup rows represent the within-group (row-wise) distribution of the LVSI category.

## Data Availability

The data presented in this study are available upon reasonable request from the corresponding author.

## Supplementary Materials

The following supporting information can be downloaded at: https://www.mdpi.com/article/10.3390/curroncol33070430/s1, Table S1: Binary logistic regression analyses using two-category LVSI classifications.
