# Supplementary material for: Substantial LVSI Is Independently Associated with Para-Aortic Nodal Metastasis in Patients Undergoing Laparoscopic Surgical Staging for Endometrial Cancer
Source: Curr Oncol. 2026 Jul 18;33(7):430. doi: 10.3390/curroncol33070430 (PMC13409510; doi:10.3390/curroncol33070430)
Supplement: Supplementary file 1 [file curroncol-33-00430-s001.zip › curroncol-4328139-supplementary.pdf]

Supplementary Table S1. Binary logistic regression analyses using two-category LVSI classifications.

LVSI Classification	Outcome	OR	95% CI	p value
Substantial vs. Negative/Focal LVSI	Overall nodal metastasis	4.94	1.62–15.09	0.005 *
Substantial vs. Negative/Focal LVSI	Para-aortic nodal metastasis	7.13	1.99–25.61	0.003 *
Any LVSI vs. Negative LVSI	Overall nodal metastasis	3.83	1.31–11.15	0.014 *
Any LVSI vs. Negative LVSI	Para-aortic nodal metastasis	4.47	1.22–16.39	0.024 *

** $p < 0.05$. OR: Odds ratio. CI: Confidence interval. LVSI: Lymphovascular space invasion. Analyses were adjusted for the same covariates as in the primary multivariable models.*
